# Impact of the Stress Hyperglycemia Ratio on In-Hospital and Long-Term Poor Prognosis in Patients with Acute Myocarditis

**DOI:** 10.31083/j.rcm2404103

**Published:** 2023-04-04

**Authors:** Yan Zhao, Jie Yang, Jing Chen, Xu Yang, Wei Zhang, Naqiang Lv, Huiqiong Tan, Yi-Da Tang

**Affiliations:** ^1^State Key Laboratory of Cardiovascular Disease, Department of Special Care Center, Fuwai Hospital, National Center for Cardiovascular Diseases, Chinese Academy of Medical Sciences and Peking Union Medical College, 100037 Beijing, China; ^2^Department of Cardiology, State Key Laboratory of Cardiovascular Disease, Fuwai Hospital, National Center for Cardiovascular Diseases, Chinese Academy of Medical Sciences and Peking Union Medical College, 100037 Beijing, China; ^3^Emergency and Critical Care Center, National Center for Cardiovascular Diseases, Fuwai Hospital, Chinese Academy of Medical Sciences and Peking Union Medical College, 100037 Beijing, China; ^4^Department of Cardiology and Institute of Vascular Medicine, Peking University Third Hospital, Key Laboratory of Molecular Cardiovascular Science, Ministry of Education, 100191 Beijing, China

**Keywords:** SHR, hyperglycemia, biomarker, acute myocarditis, prognosis

## Abstract

**Background:**

Few studies have focused on the impact of stress hyperglycemia on adverse outcomes in patients with acute myocarditis. We conducted the present study to assess the association between the stress hyperglycemia ratio (SHR) and poor prognosis in patients with acute myocarditis.

**Methods:**

From 2006 to 2020, 185 patients with acute myocarditis were enrolled. The SHR was defined as glucose at admission divided by estimated average glucose ([(1.59 × HbA1c %) – 2.59], glycated hemoglobin [HbA1c]). Participants were divided into two groups according to their SHR values. The primary endpoint was defined as in-hospital major adverse cardiovascular events (MACE), including death, heart transplantation, the need for mechanical circulatory support (MCS), and transfer to the intensive care unit (ICU). The secondary endpoint was defined as long-term MACE.

**Results:**

Subjects in the higher SHR group had more serious conditions, including lower systolic blood pressure, higher heart rate, higher white blood cell count, higher levels of alanine transaminase, troponin I, and C-reactive protein, and worse cardiac function. Multivariate logistic analysis showed that SHR >1.12 (hazard ratio (HR): 3.946, 95% confidence interval (CI): 1.098–14.182; *p* = 0.035) was independently associated with in-hospital MACE in patients with acute myocarditis. Kaplan-Meier survival analysis and multivariate Cox analysis suggested that an SHR >1.39 (HR: 1.931, 95% CI: 0.323–2.682; *p* = 0.895) was not significantly associated with long-term prognosis.

**Conclusions:**

SHR was independently associated with in-hospital adverse outcomes in patients with acute myocarditis but not with long-term prognosis.

## 1. Introduction 

Stress hyperglycemia, which is mediated by inflammation and neuroendocrine disorders, is usually accompanied by acute critical diseases and is closely associated with poor prognosis [1, 2]. There is no consensus on the diagnostic criteria for stress hyperglycemia, especially for patients with known diabetes mellitus (DM), which creates a barrier to the further study of its epidemiology, pathophysiology, and mechanism of adverse outcomes. Recently, Roberts *et al. * [3] proposed a novel marker, the stress hyperglycemia ratio (SHR; calculated from glucose at admission and estimated chronic average glucose), and suggested that it could predict adverse outcomes for patients with critical illnesses regardless of DM state. Subsequently, many researchers explored the influence of the SHR on adverse events in patients with different critical diseases, including acute coronary syndrome [4], acute myocardial infarction [5, 6], heart failure [7], stroke [8, 9], and COVID-19 [10]. Myocarditis is a critical infectious inflammatory or noninfectious inflammatory disease throughout life [11, 12]. In view of the acute severe inflammatory response, we hypothesized that the SHR is closely associated with adverse outcomes in patients with acute myocarditis. We conducted the present study to assess the association between the SHR and poor prognosis in patients with acute myocarditis.

## 2. Methods

### 2.1 Study Design and Population

This single-center, retrospective, observational study was performed at Fuwai Hospital (National Center of Cardiovascular Diseases, Beijing, China). From August 1, 2006, to March 31, 2020, a total of 269 patients who were clinically diagnosed with acute myocarditis were screened. The clinical diagnosis of acute myocarditis was in accordance with Caforio *et al*. [13], and patients meeting two or more of the following five criteria were included: (1) clinical presentations (within 3 months): chest pain, dyspnea, heart failure, syncope, palpitation, unexplained cardiogenic shock, or aborted sudden cardiac death; (2) newly abnormal electrocardiography (ECG) or Holter features; (3) elevated myocardial injury biomarkers, namely, troponin I (TnI); (4) dysfunction and structural abnormalities on echocardiographic imaging; and (5) cardiac magnetic resonance (CMR) findings meeting two or more of the Lake Louise criteria [14], namely, edema, hyperemia, and/or late gadolinium enhancement. If endomyocardial biopsy (EMB) or pathology of the heart available after heart transplantation met the revised Dallas criteria [15], the diagnosis of myocarditis was definite. Patients meeting the following criteria were excluded: (1) evidence of coronary artery stenosis ≥50%; (2) other preexisting cardiovascular disease including valvular heart disease, hypertensive heart disease, congenital heart disease or cardiomyopathy; (3) admission hemoglobin (Hb) <100 g/L; (4) admission blood glucose <3.9 mmol/L; (5) treatment with corticosteroids before admission; (6) history of ischemic or hemorrhagic stroke, renal or liver dysfunction, thyroid diseases, or malignant tumor; (7) history of erythropoietin application or blood transfusion within 30 days; and (8) missing glucose at admission, glycated hemoglobin (HbA1c), or other important laboratory test information. Ultimately, 185 patients were enrolled. **Supplementary Fig. 1** illustrates the process of enrollment.

The electronic medical records of the patients were reviewed by trained attendings. Clinical information, including demographics, medical history, coexisting diseases, physical examination, laboratory test findings, treatment regimen, and in-hospital adverse outcomes, was collected. Diabetes mellitus was diagnosed if the patient had a previous diagnosis of diabetes, used oral hypoglycemic agents or insulin, or had a measured value of HbA1c exceeding 6.5%. The estimated average glycemic level was calculated with the following formula: estimated average glucose (mmol/L) = [(1.59 × HbA1c %) – 2.59], derived from Nathan *et al*. [16]. The SHR was defined as glucose at admission divided by estimated average glucose. Participants were divided into two groups according to the optimal cutoff value of the SHR evaluated by receiver operating characteristic (ROC) analysis: the low SHR group (SHR ≤1.12, n = 111) and the high SHR group (SHR >1.12, n = 74).

During hospitalization, all patients were treated based on the recommended strategy for myocarditis [13]. Stable patients with left ventricular dysfunction received the recommended heart failure treatment. Patients with severe heart failure or cardiogenic shock were treated with inotropes and mechanical circulatory support (MCS). MCS included intra-aortic balloon pump (IABP), venous-arterial extracorporeal membrane oxygenation (va-ECMO), or a combination of IABP and va-ECMO.

### 2.2 Glycemic Status Tests

Glucose at admission was measured on the day the patient was hospitalized, and HbA1c levels were assayed between 1 and 3 days after admission. The blood samples were collected into tubes coated with EDTA-anticoagulant and centrifuged. Serum glucose was measured in the core laboratory of Fuwai Hospital using a LABOSPECT 008 system (Hitachi, Tokyo, Japan), and the HbA1c value was measured with high-performance liquid chromatography (Tosoh G8 HPLC Analyzer, Tosoh Bioscience, Tokyo, Japan).

### 2.3 Follow-up and Outcomes

After discharge, the patients were followed up by telephone interview, outpatient visits, or correspondence. All events were checked and confirmed by an independent group of trained clinical physicians. We defined the primary endpoint as in-hospital major adverse cardiovascular events (MACE), including (1) death; (2) heart transplantation; (3) a need for MCS to maintain hemodynamic stability; and (4) transfer to the intensive care unit (ICU) due to a worsening condition. The secondary endpoint was defined as long-term MACE, including (1) all-cause death; (2) heart transplantation; (3) recorded sustained ventricular arrhythmia (>30 s); (4) heart failure requiring hospitalization; and (5) myocarditis relapse.

### 2.4 Statistical Analysis

Continuous variables are described as the mean ± standard deviation (SD) or median (interquartile range) according to the results of normality tests. Categorical variables are presented as quantities and percentages. Differences between the groups were compared by Student’s *t* test or the Mann–Whitney *U* test for continuous variables and the Pearson χ^2^ test or Fisher’s exact test for categorical variables. Univariate and multivariate logistic regression and Cox proportional hazards analyses were performed to identify risk factors predicting in-hospital and long-term MACE, respectively. The confounding factors selected in the multivariate Cox analysis model included age, sex, the variables that were significantly associated with prognosis in univariate analysis, and factors that had ever been reported to be associated with MACE or might affect glucose status (coexisting diabetes mellitus, QRS duration >120 ms, creatinine, left ventricular ejection fraction (LVEF), etc.). In addition, Kaplan–Meier (K-M) survival analyses and the log-rank test were used to compare the event-free survival between the two groups. The ability of the SHR to predict MACE was assessed by receiver operating characteristic (ROC) analysis and was quantified by the area under the ROC curve (AUC), in which a value of 1.0 indicates perfect ability and a value of 0.5 indicates no ability. Analyses were performed with SPSS statistics (version 26.0, IBM Corp., Chicago, IL, USA). The K-M and ROC curves were drawn with GraphPad Prism (version 5.0, Dotmatics, Boston, MA, USA). All analyses were two tailed, and *p* values < 0.05 were considered indicative of statistical significance.

## 3. Results

### 3.1 Patient Population and Clinical Presentation

The baseline characteristics of the study population are reported in Table 1. A total of 185 patients with available SHR data were included in the analysis. The population was divided into two groups according to SHR (Table 1). The average age of the patients was 30.68 ± 12.73 years, and 132 (71.4%) patients were men. Patients in the high SHR group (SHR >1.12) were older than those in the low SHR group (SHR ≤1.12). There was no significant difference in the percentage of males, body mass index (BMI), clinical symptoms, or the prevalence of comorbidities between the two groups. Patients with higher SHR had significantly lower systolic blood pressure and higher heart rate. On ECG, patients with higher SHR had higher incidence rates of sinus tachycardia, complete atrioventricular block, and bundle-branch block, although the incidence rates of supraventricular tachycardia and sustained ventricular tachycardia were not significantly different. In addition, we found that subjects in the higher SHR group had more obvious abnormalities in laboratory test results, including higher white blood cell count, lower hemoglobin, worse liver function, and higher levels of troponin I, C-reactive protein (CRP), and admission glucose. Patients with higher SHR also had a thicker intraventricular septum and lower LVEF, and patients with LVEF <50% accounted for 41.9% of the study population. The medication regimen was not significantly different in the use of β-blockers, angiotensin-converting enzyme inhibitors/angiotensin II receptor blockers (ACEIs/ARBs), or aldosterone antagonists between the two groups. Subjects with higher SHR were more likely to require inotropic drugs and invasive life support devices (IABP, ECMO, ventilator, continuous venovenous hemofiltration (CVVH), and temporary pacing) to maintain hemodynamic stability.

**Table 1.** **Baseline characteristics of the study population grouped by SHR levels**.

		Total (n = 185)	SHR ≤1.12 (n = 111)	SHR >1.12 (n = 74)	*p* value
Demographics					
	Age (years)	30.68 ± 12.73	28.88 ± 12.03	33.38 ± 13.34	0.018
	Male, n (%)	132 (71.4)	84 (75.7)	48 (64.9)	0.111
	BMI (kg/$m^{2}$)	23.93 ± 4.23	23.90 ± 4.52	23.98 ± 3.78	0.905
Comorbidities and NYHA class					
	Hypertension, n (%)	11 (5.9)	8 (7.2)	3 (4.1)	0.530
	Diabetes mellitus, n (%)	5 (2.7)	2 (1.8)	3 (4.1)	0.390
	Dyslipidemia, n (%)	16 (8.6)	9 (8.1)	7 (9.5)	0.749
	NYHA III or IV (%)	58 (31.4)	25 (22.5)	33 (44.6)	0.002
Clinical presentation, n (%)					
	Chest pain	78 (42.2)	48 (43.2)	30 (40.5)	0.715
	Dyspnea	63 (34.1)	35 (31.5)	28 (37.8)	0.375
	Syncope	16 (8.6)	8 (7.2)	8 (10.8)	0.393
Vital signs at admission					
	Systolic blood pressure (mmHg)	111.30 ± 18.13	115.17 ± 16.92	105.54 ± 18.46	<0.001
	Diastolic blood pressure (mmHg)	68.11 ± 11.65	68.47 ± 11.49	67.58 ± 11.94	0.612
	Heart rate (beats/minute)	84.29 ± 18.56	80.32 ± 15.34	90.24 ± 21.30	0.001
Electrocardiogram at admission					
	Normal, n (%)	58 (31.4)	44 (39.6)	14 (18.9)	0.003
	QRS interval (ms)	100.61 ± 26.41	98.40 ± 25.34	103.92 ± 27.78	0.165
	QTc interval (ms)	438.33 ± 42.54	438.25 ± 40.14	438.46 ± 46.19	0.974
	QRS interval >120 ms, n (%)	26 (14.1)	13 (11.7)	13 (17.6)	0.262
	QTc interval >460 ms, n (%)	47 (25.4)	27 (24.3)	20 (27.0)	0.679
Arrhythmia, n (%)					
	Sinus tachycardia	42 (22.7)	14 (12.6)	28 (37.8)	<0.001
	Supraventricular tachycardia	11 (5.9)	4 (3.6)	7 (9.5)	0.119
	Sustained VT/VF	13 (7.0)	6 (5.4)	7 (9.5)	0.291
	complete AVB	17 (9.2)	5 (4.5)	12 (16.2)	0.007
	Bundle-branch block	27 (14.6)	11 (9.9)	16 (21.6)	0.027
Laboratory tests at admission					
	White blood cell (×${\mathrm{10}}^{9}$/L)	7.66 (6.15–10.77) *	7.26 (5.63–8.77) *	9.65 (6.92–12.11) *	<0.001
	Hemoglobin (g/L)	1142.00 (131.00–152.00) *	1143.00 (133.00–152.00) *	1135.50 (127.50–149.25) *	0.045
	ALT (IU/L)	43.00 (25.00–81.50) *	37.00 (21.00–66.00) *	54.00 (29.75–167.75) *	0.001
	Creatinine (umol/L)	78.20 (67.31–91.59) *	77.00 (67.43–88.89) *	79.61 (66.89–101.57) *	0.137
	Troponin I (ng/mL)	1.68 (0.26–5.54) *	0.958 (0.07–4.83) *	3.29 (0.79–8.38) *	0.001
	CRP (mg/L)	11.00 (4.21–29.90) *	8.46 (3.40–18.60) *	21.15 (8.66–74.23) *	<0.001
	Glucose at admission (mmol/L)	6.30 (5.63–7.45) *	5.81 (5.29–6.23) *	8.15 (7.05–9.92) *	<0.001
	HbA1c (%)	5.58 ± 0.67	5.57 ± 0.44	5.60 ± 0.91	0.772
	HbA1c (mmol/mol)	37.50 ± 7.31	37.34 ± 4.79	37.72 ± 9.90	0.772
	SHR	1.05 (0.90–1.25) *	0.94 (0.84–1.02) *	1.32 (1.20–1.53) *	<0.001
Echocardiography at admission					
	Left atrium (mm)	33.80 ± 5.35	34.10 ± 5.73	33.34 ± 4.71	0.344
	LVEDD (mm)	49.41 ± 6.72	50.06 ± 7.65	48.42 ± 4.91	0.077
	Interventricular septum (mm)	9.22 ± 1.74	8.93 ± 1.70	9.68 ± 1.71	0.004
	Right ventricular (mm)	21.42 ± 3.49	21.82 ± 3.56	20.81 ± 3.30	0.062
	LVEF (%)	54.48 ± 13.67	56.60 ± 13.81	51.27 ± 12.89	0.009
	LVEF <50%, n (%)	56 (30.3)	25 (22.5)	31 (41.9)	0.005
	CMR performed, n (%)	126 (68.1)	70 (63.1)	56 (75.7)	0.071
Medications					
	β-Blockers, n (%)	143 (77.3)	89 (80.2)	54 (73.0)	0.252
	ACEIs/ARBs, n (%)	85 (45.9)	54 (48.6)	31 (41.9)	0.366
	Aldosterone antagonists, n (%)	43 (23.2)	26 (23.4)	17 (23.0)	0.943
	Inotropic drugs	44 (23.8)	14 (13.1)	30 (40.5)	<0.001
Life support treatment					
	IABP, n (%)	16 (8.6)	4 (3.6)	12 (16.2)	0.003
	ECMO, n (%)	6 (3.2)	0 (0.0)	6 (8.1)	0.004
	Ventilator, n (%)	12 (6.5)	1 (0.9)	11 (14.9)	<0.001
	CVVH, n (%)	6 (3.2)	1 (0.9)	5 (6.8)	0.038
	Temporary pacing, n (%)	13 (7.0)	2 (1.8)	11 (14.9)	0.001

Data are expressed as mean ± SD, medians with interquartile ranges * or n (%). BMI, body mass index; VT/VF, ventricular tachycardia/ventricular fibrillation; AVB, atrioventricular block; ALT, alanine transaminase; CRP, C reactive protein; SHR, stress hyperglycemia ratio; LVEDD, left ventricular end-diastolic diameter; LVEF, left ventricular ejection fraction; CMR, cardiac magnetic resonance; ACEIs/ARBs, angiotensin-converting enzyme inhibitors/angiotensin II receptor blockers; IVIG, intravenous immunoglobulins; IABP, intra-aortic balloon pump; ECMO, arteriovenous extracorporeal membrane oxygenation; CVVH, continuous venovenous hemofiltration.

### 3.2 Etiology of Acute Myocarditis

A total of 28 (15.1%) patients underwent EMB. Among them, immunopathology findings showed lymphocyte myocarditis in 13 patients (46.4%), giant cell myocarditis in 3 patients (10.7%), and eosinophilic myocarditis in 2 patients (7.1%).

### 3.3 ROC Curve Analysis and Predictive Value for In-Hospital and Long-Term MACE

**Fig. 1.**
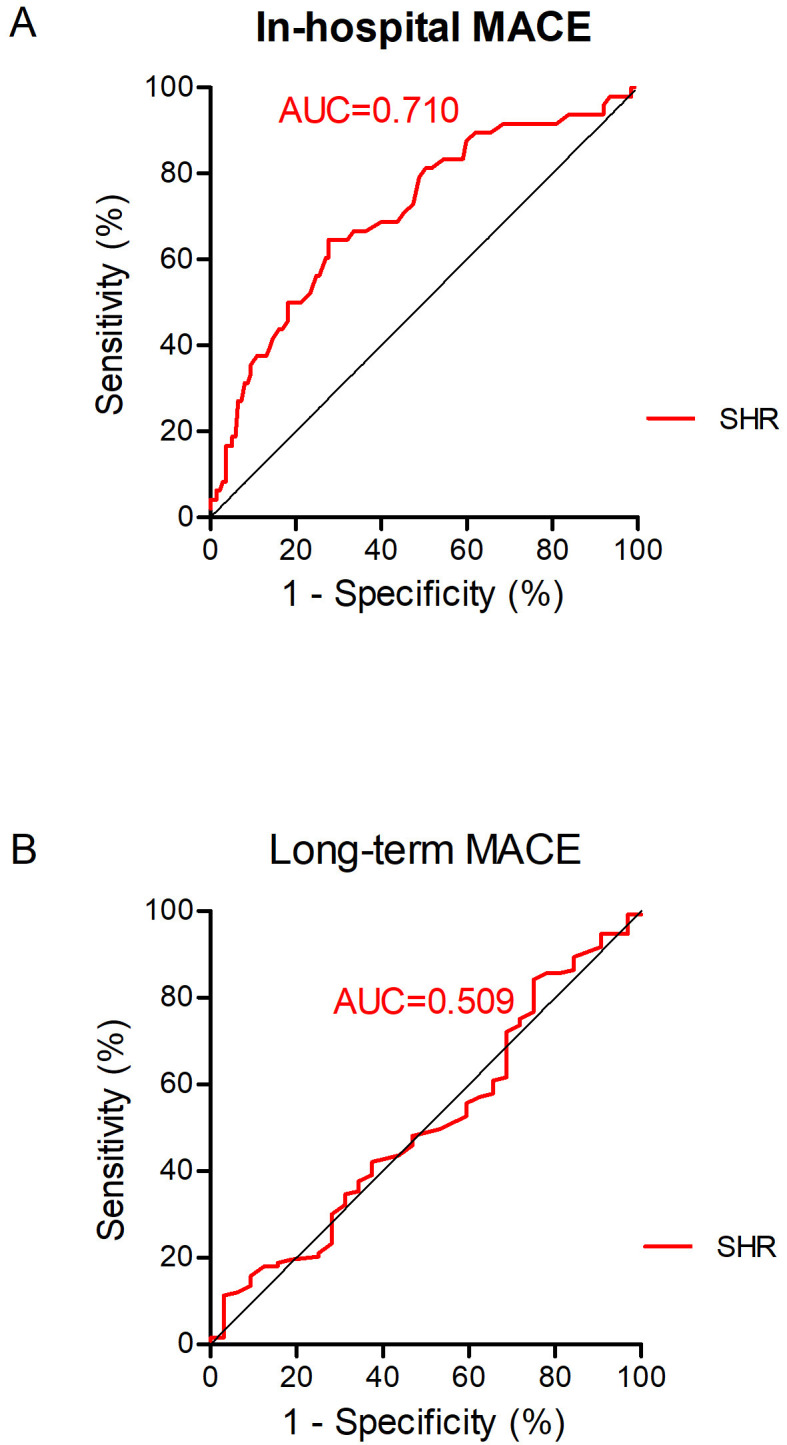
**Receiver operating characteristic (ROC) curve of the ability of SHR to predict in-hospital MACE (A) and long-term MACE (B) in patients with acute myocarditis**. In predicting in-hospital MACE including death, heart transplantation, mechanic circulatory support, and need to transfer to ICU, the area under the curve (AUC) for SHR was 0.710, with sensitivity of 64.58% and specificity of 72.26%. In predicting long-term MACE including deaths, heart transplantations, sustained ventricular tachycardias (>30 s), rehospitalization for heart failure, and myocarditis relapse, the AUC for SHR was 0.509, with sensitivity of 25.00% and specificity of 84.21%. MACE, major adverse cardiac events; SHR, stress hyperglycemia ratio.

To assess the predictive value of the SHR in the outcomes of patients with acute myocarditis, ROC curves for the SHR were generated. In predicting in-hospital MACEs, including death, heart transplantation, MCS, and transfer to the ICU, the sensitivity and specificity of the SHR were 64.58% and 72.26%, respectively (AUC = 0.710, optimal cutoff value: 1.12) (Fig. 1A). In predicting long-term MACEs, the sensitivity and specificity of the SHR were 25.00% and 84.21%, respectively (AUC = 0.509, optimal cutoff value: 1.39) (Fig. 1B).

### 3.4 Prognostic Value of the SHR in the Prognosis of Acute Myocarditis

A total of 165 patients had complete follow-up information, and there was no significant difference in baseline characteristics between the patients with follow-up (n = 165) and those lost to follow-up (n = 20), except that the corrected QT (QTc, QT means the Interval from the beginning of the Q wave to the end of the T wave on the electrocardiogram) intervals of those lost to follow-up were longer (**Supplementary Table 1**). In-hospital MACE occurred in 48 patients (25.9%) and included 9 deaths (5.5%), 2 heart transplantations (1.1%), 5 MCS (2.7%), and 32 transfers to the ICU (17.3%). After a median follow-up of 3.9 years (interquartile range 2.3 years, 6.6 years), long-term MACE had occurred in 32 patients (19.4%) and included 10 deaths (6.1%), 3 heart transplantations (1.8%), 3 sustained ventricular arrhythmias (1.8%), 7 heart failure hospitalizations (4.2%), and 9 recurrences of myocarditis (5.5%).

**Fig. 2.**
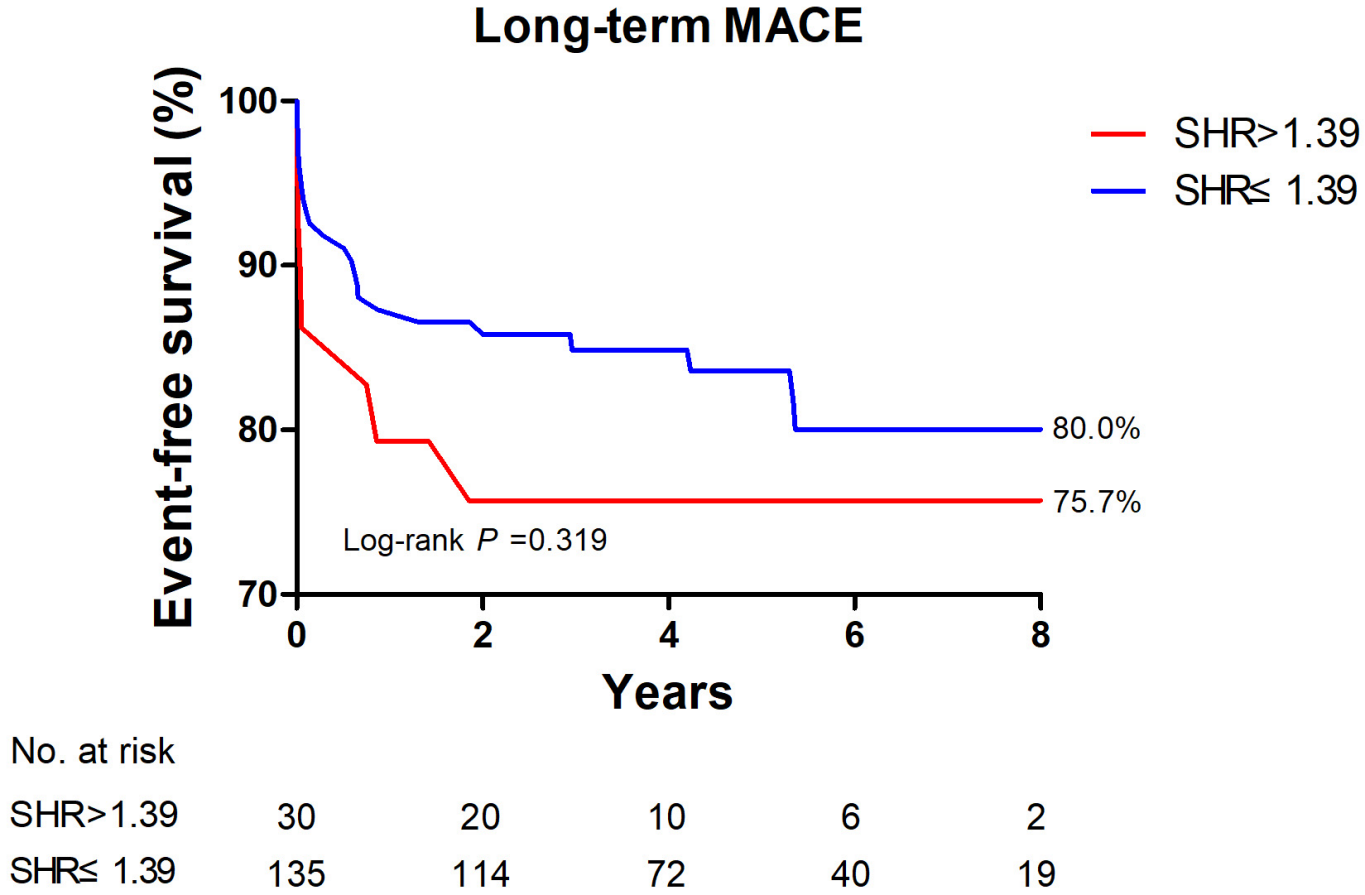
**Long-term MACE-free survival of patients with acute myocarditis, with SHR >1.39 and ≤1.39**. There was no significant difference in the two survival curves. Long-term MACE included deaths, heart transplantations, rehospitalization for heart failure, and sustained ventricular arrhythmias (>30 s), and myocarditis relapse. MACE, major adverse cardiac events; SHR, stress hyperglycemia ratio.

K-M survival analysis showed that there was no significant difference in the incidence of long-term MACE between the two groups divided around the SHR cutoff of 1.39 (Fig. 2; log-rank *p* = 0.319), although patients with SHR >1.39 had a tendency to suffer from more long-term MACEs within the first two years.

To determine whether the SHR was an independent predictor of short-term and long-term adverse outcomes, logistic and Cox regression analyses were performed (Table 2 and** Supplementary Table 2**). For the primary endpoint (in-hospital MACE), multivariate logistic analysis showed that SHR >1.12 (hazard ratio [HR]: 3.946, 95% confidence interval [CI]: 1.098–14.182; *p* = 0.035), baseline LVEF (HR: 0.887, 95% CI: 0.844–0.932; *p *< 0.001), C-reactive protein level (HR: 1.021, 95% CI: 1.009–1.032; *p *< 0.001), and alanine transaminase >120 IU/L (HR: 5.566, 95% CI: 1.347–22.997; *p* = 0.018) were independent predictors. For the secondary endpoint (long-term MACE), multivariate Cox analysis demonstrated that BMI (HR: 0.824, 95% CI: 0.744–0.912; *p <* 0.001), diabetes mellitus (HR: 6.727, 95% CI: 1.231–36.756; *p* = 0.028), creatinine level (HR: 1.007, 95% CI: 1.002–1.012; *p* = 0.007), troponin I level (HR: 1.019, 95% CI: 1.001–1.037; *p* = 0.035), and right ventricular diameter (HR: 1.185, 95% CI: 1.054–1.332; *p* = 0.004) were independent predictors. According to the above results, the SHR level was an independent predictive factor for in-hospital MACE but not for long-term prognosis in patients with acute myocarditis.

**Table 2.** **Univariate and Multivariate Logistic Analysis for In-hospital MACE**.

		HR	95% CI	*p *value
Univariate regression				
	Age, year	1.036	1.010–1.063	0.006
	Gender	2.893	1.444–5.795	0.003
	BMI, kg/$m^{2}$	0.949	0.876–1.029	0.206
	Diabetes	4.500	0.729–27.793	0.105
	QRS interval >120 ms	5.207	2.187–12.394	<0.001
	WBC at admission, ×${\mathrm{10}}^{9}$/L	1.261	1.141–1.393	<0.001
	ALT >120 IU/L	11.062	4.568–26.784	<0.001
	Creatinine, μmol/L	1.019	1.005–1.032	0.006
	Troponin I, ng/mL	1.047	1.012–1.083	0.008
	CRP, mg/L	1.018	1.010–1.027	<0.001
	RV, mm	1.061	0.996–1.166	0.214
	LVEF at admission (%)	0.896	0.867–0.925	<0.001
	SHR >1.12	4.524	2.243–9.123	<0.001
Multivariate regression				
	Age, y	1.003	0.959–1.049	0.900
	Gender	1.728	0.174–1.923	0.372
	Diabetes	0.639	0.027–14.965	0.781
	QRS interval >120 ms	4.141	0.986–17.393	0.052
	WBC at admission, ×${\mathrm{10}}^{9}$/L	0.932	0.774–1.122	0.456
	ALT >120 IU/L	5.566	1.347–22.997	0.018
	Creatinine, μmol/L	0.998	0.984–1.013	0.833
	Troponin I, ng/mL	1.054	0.995–1.117	0.071
	CRP, mg/L	1.021	1.009–1.032	<0.001
	LVEF at admission, %	0.887	0.844–0.932	<0.001
	SHR >1.12	3.946	1.098–14.182	0.035

In-hospital MACE included death, heart transplantation, need mechanic circulatory support to maintain hemodynamic stability and transfer to ICU due to worsening of conditions during hospitalization. BMI, body mass index; WBC, white blood cell; ALT, alanine transaminase; CRP, C reactive protein; LVEF, left ventricular ventricle ejection fraction; SHR, stress hyperglycemia ratio.

### 3.5 Sensitivity Analysis

Sensitivity analysis was carried out to test the association between the SHR and adverse outcomes in patients without diabetes mellitus. The five patients diagnosed with diabetes mellitus were excluded, and both logistic and Cox regression analyses were performed (**Supplementary Tables 3,4**). The results suggested that the SHR remained an independent predictor of in-hospital adverse outcomes in patients with acute myocarditis, even for nondiabetic patients.

## 4. Discussion

This study is, the first to explore the association between the SHR and short-term and long-term prognoses in patients with acute myocarditis. The following are its two main findings: (1) Patients with a higher SHR were in more serious condition, had more complications and were more likely to need MCS to maintain hemodynamic stabilization. (2) The SHR was independently associated with in-hospital outcomes but not with long-term prognosis in patients with acute myocarditis.

Stress hyperglycemia is defined as a transient episode of hyperglycemia resulting from acute illness, which can resolve automatically after the acute disease abates in most cases [1, 17]. When the body is under stress, the neuroendocrine system is activated, including enhancement of the sympathetic nervous system and elevated levels of catecholamines, steroid hormones, inflammatory cytokines, and glucagon, which can lead to insulin resistance by accelerating the decomposition of liver glycogen and gluconeogenesis [2]. Several studies [18, 19, 20, 21, 22, 23] have showed an independent association between stress hyperglycemia and poor outcomes in patients with acute cardiovascular diseases, especially those with acute myocardial infarction. The underlying mechanisms of the negative impact of acute hyperglycemia on cardiovascular diseases may include oxidative stress, endothelial dysfunction, impaired platelet nitric oxide responsiveness, atherogenic and prothrombotic effects, proinflammatory effects, and mitochondrial impairment [2, 24, 25, 26, 27, 28, 29, 30]. In addition, acute hyperglycemia may cause a negative effect on patients with viral infection [31]. Considering that the main pathophysiological mechanism of acute myocarditis is acute inflammatory damage to cardiomyocytes, and that its main etiology is viral infection, we hypothesized that stress hyperglycemia was also associated with poor prognosis in patients with acute myocarditis. However, there are no uniform diagnostic criteria for stress hyperglycemia, and acute hyperglycemia cannot be fully reflected by glucose at admission. The chronic average glucose level, which can be estimated as estimated average glucose (mmol/L) = [(1.59 × HbA1c %) – 2.59], should not be ignored. Roberts *et al*. [3] proposed a composite index, namely, the SHR, which could balance acute admission glucose and chronic average glucose, and found that the SHR was a better predictor of in-hospital death and need for critical care than absolute hyperglycemia in patients acutely admitted to a tertiary hospital. Since then, a series of studies have suggested that the SHR is closely related to adverse outcomes in patients with various acute illnesses, including acute myocardial infarction, acute heart failure, stroke, and COVID-19. Marenzi *et al*. [5] prospectively enrolled 1553 patients with AMI from June 2010 to June 2016. Admission glucose and HbA1c were examined for all patients at the hospital, and the primary endpoint was defined as the combination of in-hospital death, cardiogenic shock, and acute pulmonary edema. The results showed that SHR ≥1.3 (odds ratio [OR]: 3.91, 95% CI: 2.83–5.42; *p *< 0.001) was independently associated with in-hospital adverse outcomes. Gao *et al*. [6] consecutively enrolled 1300 patients with ST-segment elevation myocardial infarction (STEMI) treated with percutaneous coronary intervention from January 2013 to June 2018. The study endpoint was defined as in-hospital MACE. The findings of that study indicated that the SHR was closely related to in-hospital outcomes in STEMI patients regardless of diabetic status (diabetic patients: OR: 2.45; 95% CI: 1.24–4.82; *p* = 0.010; nondiabetic patients: OR: 5.84; 95% CI: 2.50–13.66; *p *< 0.001). Carrera *et al*. [7] evaluated the association between the SHR and four-year mortality in a cohort of patients hospitalized for acute heart failure. They consecutively included 1062 patients between January 2005 and December 2012. The results showed that the SHR was negatively associated with long-term mortality (HR: 0.79, 95% CI: 0.64–0.99; *p *< 0.040). The discrepant outcomes may be explained as follows. First, the glucose level at admission of enrolled patients in Carrera *et al*.’s [7] study was relatively low, suggesting that the incidence of stress hyperglycemia may have been too low. Moreover, the impact on mortality of an imbalance between glucose at admission and chronic glucose control may have been magnified because the authors did not exclude patients with acute hypoglycemia because the proportion of diabetic patients was relatively high.

In this study, we discovered that the SHR could reflect the severity of acute myocarditis. The higher the SHR was, the higher the inflammation index, the worse the cardiac function, and the higher the incidence of MCS application, which is, to some extent, consistent with previous studies on other cardiovascular diseases [4, 32, 33]. Moreover, the SHR was an independent risk factor for in-hospital outcomes but not for long-term prognosis, although patients with SHR >1.39 had a tendency to suffer from more long-term MACE within the first two years. This phenomenon illustrates the short-term predictive value of the SHR, which was in accordance with the pathophysiological mechanism of stress hyperglycemia, namely, most cases were transient hyperglycemia and resolve themselves spontaneously. The outcomes could be partly ascribed to the length of follow-up; that is, with a longer follow-up, the association between the SHR and adverse outcomes became nonsignificant regardless of whether the correlation between diabetes mellitus and poor prognosis was significant, which is in line with previous studies [19, 34]. In the sensitivity analysis we performed to exclude patients with diabetes mellitus to avoid a potential influence of that disease, the results remained robust, suggesting that the SHR correlated with in-hospital outcomes in the overall population or in nondiabetic patients with acute myocarditis. In the future, we should emphasize the occurrence of stress hyperglycemia and glucose management, preferably with insulin, when treating patients with acute myocarditis.

## 5. Strengths and Limitations

This might be the first study to concentrate on the impact of the SHR on adverse outcomes in patients with myocarditis. The baseline characteristics were comprehensive, and the endpoints included in-hospital outcomes and long-term outcomes. One limitation is that, in view of its retrospective nature and the exclusion of subjects with unmeasured HbA1c, recall bias and selection bias might be present. The proportion patients who underwent EMB was relatively low, so many patients were diagnosed according to clinical criteria. Moreover, the glucose data after hospitalization and discharge were incomplete, which made it impossible to determine the changes in abnormal glucose metabolism.

## 6. Conclusions

The SHR was independently associated with in-hospital adverse outcomes in patients with acute myocarditis but not with long-term prognosis. More multicenter, prospective cohort studies are needed to explore its predictive value in different populations.

## Data Availability

The datasets used and/or analyzed during the current study are available from the corresponding author on reasonable request.

## Abbreviations

SHR, stress hyperglycemia ratio; LVEF, left ventricular ejection fraction; MACE, major adverse cardiac events; MCS, mechanical circulatory support; ICU, intensive care unit; OR, odds ratio; HR, hazard ratio; CI, confidence interval; ROC, receiver operating characteristic; AUC, area under the ROC curve; DM, diabetes mellitus; ECG, electrocardiography; TnI, troponin I; CMR, cardiac magnetic resonance; EMB, endomyocardial biopsy; HbA1c, glycated hemoglobin; IABP, intra-aortic balloon pump; va-ECMO, venous-arterial extracorporeal membrane oxygenation; SD, standard deviation; LVEF, left ventricular ejection fraction; K-M, Kaplan–Meier; CRP, C-reactive protein; CVVH, venovenous hemofiltration.

## Supplementary Material

Supplementary material associated with this article can be found, in the online version, at https://doi.org/10.31083/j.rcm2404103.
