# Supplementary material for: Impact of the Stress Hyperglycemia Ratio on In-Hospital and Long-Term Poor Prognosis in Patients with Acute Myocarditis
Source: Rev Cardiovasc Med. 2023 Apr 4;24(4):103. doi: 10.31083/j.rcm2404103 (PMC11273025; doi:10.31083/j.rcm2404103)
Supplement: Supplementary file 1 [file 2153-8174-24-4-103-s1.zip › 2153-8174-24-4-103-s1.docx]

Supplementary Material

Supplementary Table 1. Baseline characteristics of the patients with follow-up and lost to follow-up

|  | Follow-up group  (n = 165) | Lost to follow-up  (n = 20) | *P* Value |
| --- | --- | --- | --- |
| Demographics |  |  |  |
| Age (years) | 30.30 ± 12.67 | 33.85 ± 13.14 | 0.531 |
| Male, n (%) | 118 (71.5) | 14 (70.0) | 0.887 |
| BMI (kg/m^2^) | 23.85 ± 4.20 | 24.59 ± 4.53 | 0.634 |
| Comorbidities and NYHA class |  |  |  |
| Hypertension, n (%) | 9 (5.5) | 2 (10.0) | 0.338 |
| Diabetes mellitus, n (%) | 5 (3.0) | 0 (0) | 1.000 |
| Dyslipidemia, n (%) | 14 (8.5) | 2 (10.0) | 0.685 |
| NYHA III or IV (%) | 54 (32.7) | 4 (20.0) | 0.247 |
| Clinical presentation, n (%) |  |  |  |
| Chest pain | 72 (43.6) | 6 (30.0) | 0.244 |
| Dyspnea | 57 (34.5) | 6 (30.0) | 0.685 |
| Syncope | 15 (9.1) | 1 (50.0) | 1.000 |
| Vital signs at admission |  |  |  |
| Systolic blood pressure (mmHg) | 111.03 ± 18.73 | 114.70 ± 12.41 | 0.062 |
| Diastolic blood pressure (mmHg) | 67.96 ± 11.84 | 69.70 ± 9.76 | 0.262 |
| Heart rate (beats/minute) | 84.28 ± 17.75 | 84.30 ± 24.77 | 0.191 |
| Electrocardiogram at admission |  |  |  |
| Normal, n (%) | 49 (29.7) | 9 (45.0) | 0.164 |
| QRS interval (ms) | 101.51 ± 26.62 | 93.20 ± 23.93 | 0.620 |
| QTc interval (ms) | 437.73 ± 40.04 | 443.30 ± 60.46 | 0.005 |
| QRS interval > 120ms, n (%) | 23 (13.9) | 3 (15.0) | 1.000 |
| QTc interval > 460ms, n (%) | 41 (24.8) | 6 (30.0) | 0.617 |
| Arrhythmia, n (%) |  |  |  |
| Sinus tachycardia | 37 (22.4) | 5 (25.0) | 0.781 |
| Supraventricular tachycardia | 11 (6.7) | 0 (0) | 0.612 |
| Sustained VT/VF | 13 (7.9) | 0 (0) | 0.367 |
| complete AVB | 16 (9.7) | 1 (5.0) | 0.699 |
| Bundle-branch block | 24 (14.5) | 3 (15.0) | 1.000 |
| Laboratory tests at admission |  |  |  |
| White blood cell (×10^9^/L) | 7.68 (6.31-10.88) * | 7.41 (5.52-9.86) * | 0.372 |
| Hemoglobin (g/L) | 1141.00 (131.00-150.00) * | 1147.50 (125.75-157.25) * | 0.199 |
| ALT (IU/L) | 41.00 (24.50-77.00) * | 54.50 (28.50-84.75) * | 0.435 |
| Creatinine (μmol/L) | 78.20 (68.11-92.88) * | 77.83 (64.17-87.90) * | 0.398 |
| Troponin I (ng/ml) | 1.68 (0.25-5.54) * | 1.87 (0.26-7.96) * | 0.730 |
| CRP (mg/L) | 11.00 (4.27-28.40) * | 12.03 (2.59-55.55) * | 0.810 |
| Glucose at admission (mmol/L) | 6.33 (5.65-7.45) * | 6.00 (5.55-8.45) * | 0.611 |
| SHR | 1.05 (0.91-1.24) * | 0.99 (0.88-1.26) * | 0.536 |
| Echocardiography at admission |  |  |  |
| Left atrium (mm) | 33.79 ± 5.52 | 33.85 ± 3.70 | 0.093 |
| LVEDD (mm) | 49.62 ± 6.89 | 47.65 ± 4.99 | 0.438 |
| Interventricular septum (mm) | 9.22 ± 1.79 | 9.27 ± 1.23 | 0.260 |
| Right ventricular (mm) | 21.57 ± 3.45 | 20.21 ± 2.89 | 0.593 |
| LVEF (%) | 54.01 ± 13.87 | 58.31 ± 11.43 | 0.061 |
| LVEF < 50%, n (%) | 52 (31.5) | 4 (20.0) | 0.290 |
| CMR performed, n (%) | 114 (69.1) | 12 (60.0) | 0.410 |
| Medications |  |  |  |
| β-Blockers, n (%) | 128 (77.6) | 15 (75.0) | 0.781 |
| ACEI/ARBs, n (%) | 76 (46.1) | 9 (45.0) | 0.928 |
| Aldosterone antagonists, n (%) | 41 (24.8) | 2 (10.0) | 0.169 |
| Inotropic drugs | 40 (24.7) | 4 (21.1) | 1.000 |
| Life support treatment |  |  |  |
| IABP, n (%) | 13 (7.9) | 3 (15.0) | 0.389 |
| ECMO, n (%) | 5 (3.0) | 1 (5.0) | 0.502 |
| Ventilator, n (%) | 11 (6.7) | 1 (5.0) | 1.000 |
| CVVH, n (%) | 6 (3.6) | 0 (0) | 1.000 |
| Temporary pacing, n (%) | 13 (7.9) | 0 (0) | 0.367 |

Data are expressed as mean ± SD, medians with interquartile ranges * or n (%)

BMI, body mass index; VT/VF, ventricular tachycardia/ventricular fibrillation; AVB, atrioventricular block; ALT, alanine transaminase; CRP, C reactive protein; SHR, stress hyperglycemia ratio; LVEDD, left ventricular end-diastolic diameter; LVEF, left ventricular ejection fraction; CMR, cardiac magnetic resonance ; ACEI/ARB, angiotensin-converting enzyme inhibitors/angiotensin II receptor blockers; IVIG, intravenous immunoglobulins; IABP, intra-aortic balloon pump; ECMO, arteriovenous extracorporeal membrane oxygenation; CVVH, continuous venovenous hemofiltration; Q1-Q3, quartile 1-3.

Supplementary Table 2. Univariate and Multivariate Cox Analysis for Long-term MACE

|  | HR | 95%CI | *P* Value |
| --- | --- | --- | --- |
| **Univariate regression** |  |  |  |
| Age, y | 1.018 | 0.992-1.044 | 0.177 |
| Gender | 1.446 | 0.696-3.004 | 0.323 |
| BMI, kg/m^2^ | 0.866 | 0.786-0.955 | 0.004 |
| Diabetes | 2.848 | 0.679-11.939 | 0.152 |
| QRS interval >120ms | 2.867 | 1.325-6.202 | 0.007 |
| WBC at admission, ×10^9^/L | 1.133 | 1.041-1.232 | 0.004 |
| ALT > 120 IU | 3.445 | 1.658-7.159 | 0.001 |
| Creatinine, μmol/L | 1.009 | 1.005-1.012 | < 0.001 |
| CRP, mg/L | 1.001 | 0.992-1.009 | 0.897 |
| Troponin I, ng/mL | 1.019 | 1.004-1.035 | 0.014 |
| RV, mm | 1.187 | 1.099-1.282 | < 0.001 |
| LVEF at admission, % | 0.945 | 0.923-0.967 | < 0.001 |
| SHR > 1.39 | 1.667 | 0.749-3.713 | 0.211 |
| **Multivariate regression** |  |  |  |
| Age, y | 0.989 | 0.960-1.019 | 0.483 |
| Gender | 1.515 | 0.571-4.017 | 0.404 |
| BMI, kg/m^2^ | 0.824 | 0.744-0.912 | < 0.001 |
| Diabetes | 6.727 | 1.231-36.756 | 0.028 |
| QRS interval >120ms | 0.950 | 0.349-2.592 | 0.921 |
| WBC at admission, ×10^9^/L | 1.030 | 0.913-1.162 | 0.631 |
| ALT > 120 IU/L | 1.425 | 0.506-4.015 | 0.503 |
| Creatinine, μmol/L | 1.007 | 1.002-1.012 | 0.007 |
| Troponin I, ng/mL | 1.019 | 1.001-1.037 | 0.035 |
| RV, mm | 1.185 | 1.054-1.332 | 0.004 |
| LVEF at admission, % | 0.974 | 0.946-1.003 | 0.074 |
| SHR > 1.39 | 1.931 | 0.323-2.682 | 0.895 |

Long-term MACE included death, heart transplantation, sustained ventricular tachycardia, recorded sustained ventricular arrhythmia (> 30s), heart failure requiring hospitalization, and myocarditis relapse. BMI, body mass index; WBC, white blood cell; ALT, alanine transaminase; CRP, C reactive protein; RV, right ventricular diameter; LVEF, left ventricular ventricle ejection fraction; SHR, stress hyperglycemia ratio.

Supplementary Table 3. Univariate and Multivariate Logistic Analysis for In-hospital MACE in Patients without Diabetes

|  | HR | 95%CI | *P* Value |
| --- | --- | --- | --- |
| **Univariate regression** |  |  |  |
| Age, year | 1.033 | 1.006-1.060 | 0.015 |
| Gender | 2.684 | 1.317-5.469 | 0.007 |
| BMI, kg/m^2^ | 0.938 | 0.863-1.021 | 0.138 |
| QRS interval > 120ms | 5.645 | 2.291-13.909 | < 0.001 |
| WBC at admission, ×10^9^/L | 1.249 | 1.130-1.380 | < 0.001 |
| ALT > 120 IU/L | 11.200 | 4.571-27.441 | < 0.001 |
| Creatinine, umol/L | 1.016 | 1.003-1.029 | 0.014 |
| Troponin I, ng/mL | 1.044 | 1.010-1.079 | 0.011 |
| CRP, mg/L | 1.017 | 1.009-1.026 | < 0.001 |
| RV, mm | 1.051 | 0.995-1.157 | 0.308 |
| LVEF at admission (%) | 0.899 | 0.870-0.929 | < 0.001 |
| SHR > 1.12 | 4.585 | 2.231-9.422 | < 0.001 |
| **Multivariate regression** |  |  |  |
| Age, y | 1.002 | 0.958-1.048 | 0.934 |
| Gender | 1.696 | 0.509-5.647 | 0.389 |
| QRS interval > 120ms | 4.776 | 1.088-20.966 | 0.038 |
| WBC at admission, ×10^9^/L | 0.931 | 0.755-1.119 | 0.446 |
| ALT > 120 IU/L | 5.053 | 1.203-21.226 | 0.027 |
| Creatinine, umol/L | 0.999 | 0.985-1.013 | 0.842 |
| Troponin I, ng/mL | 1.049 | 0.991-1.110 | 0.099 |
| CRP, mg/L | 1.020 | 1.009-1.032 | 0.001 |
| LVEF at admission, % | 0.890 | 0.847-0.935 | < 0.001 |
| SHR > 1.12 | 4.084 | 1.124-14.837 | 0.033 |

In-hospital MACE included death, heart transplantation, need mechanic circulatory support to maintain hemodynamic stability and transfer to ICU due to worsening of conditions during hospitalization. BMI, body mass index; WBC, white blood cell; ALT, alanine transaminase; CRP, C reactive protein; LVEF, left ventricular ventricle ejection fraction; SHR, stress hyperglycemia ratio.

Supplementary Table 4. Univariate and Multivariate Cox Analysis for Long-term MACE in Patients without Diabetes

|  | HR | 95%CI | *P* Value |
| --- | --- | --- | --- |
| **Univariate regression** |  |  |  |
| Age, y | 1.015 | 0.988-1.042 | 0.279 |
| Gender | 1.158 | 0.530-2.534 | 0.713 |
| BMI, kg/m^2^ | 0.848 | 0.764-0.940 | 0.002 |
| QRS interval > 120ms | 2.855 | 1.270-6.421 | 0.011 |
| WBC at admission, ×10^9^/L | 1.124 | 1.030-1.227 | 0.009 |
| ALT > 120 IU | 3.252 | 1.520-6.959 | 0.002 |
| Creatinine, umol/L | 1.009 | 1.006-1.012 | < 0.001 |
| CRP, mg/L | 1.000 | 0.990-1.009 | 0.938 |
| Troponin I, ng/mL | 1.019 | 1.004-1.035 | 0.015 |
| RV, mm | 1.191 | 1.101-1.288 | < 0.001 |
| LVEF at admission, % | 0.948 | 0.926-0.971 | < 0.001 |
| SHR > 1.39 | 1.569 | 0.673-3.658 | 0.297 |
| **Multivariate regression** |  |  |  |
| Age, y | 0.988 | 0.958-1.019 | 0.444 |
| Gender | 1.122 | 0.401-3.143 | 0.826 |
| BMI, kg/m^2^ | 0.812 | 0.732-0.901 | < 0.001 |
| QRS interval > 120ms | 0.851 | 0.282-2.568 | 0.774 |
| WBC at admission, ×10^9^/L | 1.030 | 0.910-1.166 | 0.641 |
| ALT > 120 IU/L | 1.203 | 0.388-3.727 | 0.749 |
| Creatinine, umol/L | 1.008 | 1.002-1.013 | 0.005 |
| Troponin I, ng/mL | 1.022 | 1.003-1.040 | 0.021 |
| RV, mm | 1.194 | 1.053-1.354 | 0.006 |
| LVEF at admission, % | 0.974 | 0.946-1.003 | 0.074 |
| SHR > 1.39 | 1.130 | 0.360-3.549 | 0.835 |

Long-term MACE included death, heart transplantation, sustained ventricular tachycardia, recorded sustained ventricular arrhythmia (> 30s), heart failure requiring hospitalization, and myocarditis relapse. BMI, body mass index; WBC, white blood cell; ALT, alanine transaminase; CRP, C reactive protein; RV, right ventricular diameter; LVEF, left ventricular ventricle ejection fraction; SHR, stress hyperglycemia ratio.


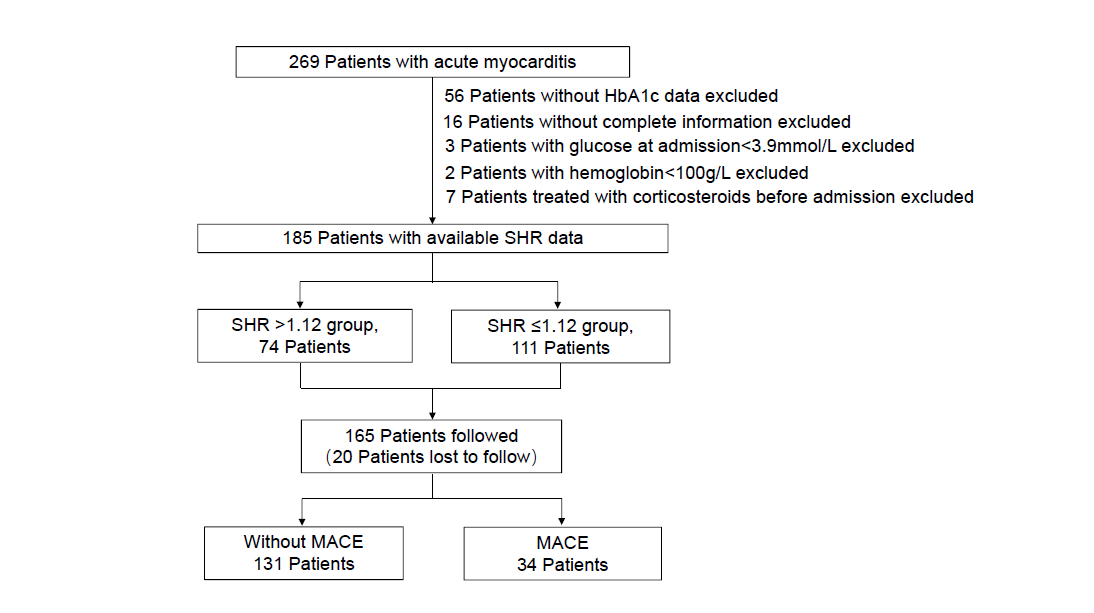


**Figure 1.** The flow chart of the enrollment of 185 adult patients with acute myocarditis from the overall population with acute myocarditis.
